# U.S. EPA oversight of pesticide traits in genetically modified plants and recent biotechnology innovation efforts

**DOI:** 10.3389/fpls.2023.1126006

**Published:** 2023-02-23

**Authors:** Michael Mendelsohn, Amanda A. Pierce, Wiebke Striegel

**Affiliations:** Emerging Technologies Branch, Biopesticides and Pollution Prevention Division, Office of Pesticide Programs, United States Environmental Protection Agency, Washington, DC, United States

**Keywords:** genome editing, plant biotechnology, plant protection, pesticide, regulatory policy, environmental protection

## Abstract

Before pesticides can be sold in the United States, the United States Environmental Protection Agency (EPA) must evaluate them thoroughly to ensure that they meet U.S. federal pesticide registration standards for human health and the environment. EPA considers pesticidal substances produced and used in plants as pesticides and defined them in the regulations as “plant-incorporated protectants” (PIPs). PIPs that are created through conventional breeding are exempted from registration requirements, while those created through biotechnology require individual assessments and approval by EPA before they can be distributed or used. This currently includes PIPs that are identical to those that could be moved through conventional breeding but are created through biotechnology (e.g., through genome editing or *via* precision breeding techniques). EPA proposed an exemption in October 2020 to allow certain PIPs created through biotechnology to be exempt from EPA requirements for pesticides where those PIPs: 1) pose no greater risk than PIPs that EPA has already exempted, and 2) could have otherwise been created through conventional breeding.

## Introduction

The U.S. Federal government issued its Coordinated Framework for Biotechnology Products in 1986 and updated this framework in 1992 and 2017. The framework describes a comprehensive regulatory policy for ensuring the safety of biotechnology products and the allocation and coordination of oversight responsibilities under the relevant statutes and among the U.S. Federal agencies. Under the Coordinated Framework, EPA regulates PIPs as pesticides.

Before pesticides can be sold in the United States, the United States Environmental Protection Agency (EPA) must evaluate them thoroughly to ensure that they meet U.S. federal pesticide registration standards to protect human health and the environment. EPA grants a “registration” or license that permits a pesticide’s distribution, sale, and use only after the company meets scientific and regulatory requirements. Under the Federal Insecticide, Fungicide, and Rodenticide Act (FIFRA), EPA evaluates pesticides including PIPs for their effects on the environment and human health and regulates their development, sale, distribution, and use. Under the Federal Food, Drug and Cosmetic Act (FFDCA), EPA evaluates PIPs that are proposed for use in food or feed. In its assessment the Agency considers all anticipated dietary exposures, as well as residential and other outdoor uses.

EPA considers pesticidal substances produced and used in plants to be pesticides and defines them as “plant-incorporated protectants” (PIPs) at Title 40 of the Code of Federal Regulations (40 CFR) § 174.3. PIPs include not only the pesticidal substances produced by plants but also the genetic material necessary for the plant to produce that substance. For example, a gene for a specific pesticidal protein, such as the Cry1Ab protein derived from the soil microorganism *Bacillus thuringiensis*, is introduced into the genome of a plant. The plant then produces from that gene the pesticidal protein that protects the plant from plant-feeding pests. Here, both the Cry1Ab protein and its genetic material in the plant are regulated by EPA as a pesticide.

## EPA’s 2001 exemption for PIPs moved through conventional breeding does not include PIPs developed through biotechnology

Plants naturally produce substances that have pesticidal properties. When EPA developed its regulations for PIPs (Federal Register, 2001), it determined that these pesticidal substances naturally produced by plants are PIPs when intended for pesticide use in the plant. However, EPA did not intend to regulate PIPs that naturally occur in plants, which had long been selected for in conventional plant breeding. Thus, when EPA promulgated its regulations for genetically engineered PIPs in 2001, it also published exemptions for PIPs created from sexually compatible plants moved through conventional breeding (40 CFR 174.25, 174.705, and 174.508) with the exception that adverse effects reporting requirements still apply (40 CFR 174.71). These exemptions reflect the history of safe use of PIPs in conventional breeding.

Because the “conventional breeding” definition that formed the basis of the 2001 exemptions specifically excludes PIPs developed through biotechnology (40 CFR 174.3), PIPs that are identical to those that could be moved through conventional breeding but are created through biotechnology currently must be registered. This includes those PIPs that are created through gene editing. When the 2001 rule was promulgated, precision breeding techniques such as genome editing were unavailable and EPA determined that additional criteria needed to be developed that would allow the Agency to include PIPs developed through biotechnology that are found in sexually compatible plants in the exemption. Thus, EPA issued a supplemental proposal entitled: “Plant- Incorporated Protectants (PIPs); Exemption for Those Derived Through Genetic Engineering From Sexually Compatible Plants.” This proposed rule was ultimately withdrawn in 2018 (Federal Register, 2018) because the Agency determined that to exempt PIPs created through genetic engineering from sexually compatible plants, exemption criteria needed to be developed to reflect advances in genetics and molecular biology since the 2001 proposal. Consequently, EPA indicated that to pursue a future exemption, the Agency would issue a new proposed rule based on the types of products possible to create with the current state of scientific advances rather than issue a final rule based on previous proposals.

## Biotechnology innovation efforts within the United States Government and at EPA

EPA indicated in the “National Strategy for Modernizing the Regulatory System for Biotechnology Products” (OSTP, 2016) that it intended to clarify its approach to pesticidal products derived from genome editing.

On October 29, 2018, FDA issued its “Plant and Animal Biotechnology Innovation Action Plan” where it indicated FDA’s intent to develop guidance for industry on how current FDA regulatory policy for foods derived from new plant varieties applies to foods produced using genome editing.

On June 11, 2019, Executive Order 13874 on “Modernizing the Regulatory Framework for Agricultural Biotechnology Products” was issued. Section 4(b) of that Executive Order directed the U.S. Department of Agriculture (USDA), EPA, and the Food and Drug Administration (FDA) “to the extent consistent with law and the principles set forth in section 3” of the order to “use existing statutory authority, as appropriate, to exempt low-risk products of agricultural biotechnology from undue regulation.”

Subsequently on May 18, 2020, USDA revised its plant pest biotechnology regulations at 7 CFR part 340. In that rule, USDA amended its regulations in response to advances in genetic engineering as well as USDA’s understanding of the associated plant pest risk posed by genetically engineered organisms.

EPA then proposed a rule on October 9, 2020 to exempt certain PIPs based on sexually compatible plants created through biotechnology. It should be noted that EPA and USDA use the term “conventional breeding” in the context of their own regulations.

On September 12, 2022, Executive Order 14081 on Biotechnology and Biomanufacturing was issued. Under this new Executive Order, EPA, USDA and FDA are working to improve the clarity and efficiency of regulatory processes for biotechnology products. EPA is currently working to finalize its proposed exemption for certain PIPs.

## EPA’s proposed exemption for PIPs based on sexually compatible plants created through biotechnology

Advances in genome editing (e.g., the CRISPR-Cas nuclease system, meganucleases, zinc-finger nucleases, and transcription activator-like effector nucleases) allow for targeted, rapid, and precise changes to chromosomes of living cells (NASEM, 2017). These technologies allow editing of the genome in a way that the resulting genes can be indistinguishable from those found in a plant created through conventional breeding.

EPA’s proposed rule reflects these scientific advances and would allow certain PIPs created through biotechnology to be exempt under the pesticide licensing and use law (FIFRA) and the law used to regulate pesticide residues in food and feed (FFDCA), in cases where those PIPs: 1) pose no greater risk than PIPs that EPA has already exempted, and 2) could have otherwise been created through conventional breeding. To further describe the types of PIPs that would meet these criteria, the Agency proposed new definitions to limit the pesticidal substances that would fit under the exemption to those found in plants that are sexually compatible with the recipient plant, i.e., definitions for “native gene” and “native allele.” “Native allele” is proposed to mean a variant of a native gene that is identified in the genetic diversity of plants sexually compatible with the recipient plant. “Native gene” is proposed to mean a gene that is identified in the recipient plant or plants sexually compatible with the recipient plant; and has never been derived from a source that is not sexually compatible with the source plant.” Through these definitions, the proposal also excludes use of transgenes that could be moved between sexually compatible plants through conventional breeding. For example, a Cry1Ab protein from *B. thuringiensis* that was engineered into a source plant would not qualify as a native gene to be used in a recipient plant since *B. thuringiensis* and the recipient plant are not sexually compatible. By limiting the pesticidal substances to only those that are found in plants sexually compatible with the recipient plant, EPA can rely on the history of safe use associated with conventional breeding to conclude negligible risk of novel exposures or hazards.

The proposal allows developers to modify an existing gene to create a “native allele” or insert a “native gene.” This allows for modifications within the coding region of an existing native gene in a plant to create a native allele, and insertion of a native gene into non-genic regions of the genome.

The proposal also allows developers to make modifications in the expression level of an existing native gene and for the reduction or elimination of a substance that is itself not pesticidal, but its absence has a pesticidal effect.

Lastly, the proposal included a process to determine the eligibility for exemption: 1) a developer may submit either a self-determination letter, and/or 2) request EPA confirmation that their PIP meets the criteria for exemption.

## What will the final exemption for PIPs based on sexually compatible plants created through biotechnology look like and when will it be final?

EPA received a total of 8,120 comments in response to its proposed rule. Of those, 28 were unique and one of those unique comments was supported by 8,093 co-signers. Many commenters supported EPA’s effort to exempt certain PIPs that are created through newer biotechnology techniques. However, commenters across industry, trade, and academia felt that the proposed exemptions could be broadened. Some commenters found the proposal to be too permissive and recommended specific modifications.

EPA is taking these comments into consideration and is in the process of developing a final rule to exempt PIPs based on sexually compatible plants created through biotechnology.

## Author contributions

All authors listed have made a substantial, direct, and intellectual contribution to the work, and approved it for publication.
